# Substantial Depletion of Vicine, Levodopa, and Tyramine in a Fava Bean Protein-Based Nutritional Product

**DOI:** 10.1155/2021/6669544

**Published:** 2021-01-29

**Authors:** Paul W. Johns, Steven R. Hertzler

**Affiliations:** Abbott Laboratories, Abbott Nutrition Division, 3300 Stelzer Road, Columbus, OH, USA 43219

## Abstract

A commercial fava bean protein isolate and a liquid nutritional product formulated with it were tested by validated HPLC methods for the favism-associated pyrimidine glycoside vicine, the dopamine precursor levodopa, and the biogenic amine tyramine. The vicine, levodopa, and tyramine concentrations in the protein isolate—306, 13.3, and <0.5 mg/kg, respectively—when expressed on a protein basis—34, 1.5, and <0.06 mg/100 g protein, respectively—were at least 96% lower than the vicine, levodopa, and tyramine (protein-based) concentrations reported for fava beans (≥900, ~200, and ~4 mg/100 g protein, respectively). This was also true for the vicine (13 mg/kg or 22 mg/100 g protein), levodopa (≤0.17 mg/kg or ≤0.3 mg/100 g protein), and tyramine (0.08 mg/kg or 0.14 mg/100 g protein) concentrations in the nutritional product. On the basis of these data, one serving (11 fl. oz.) of the nutritional product would deliver approximately 5 mg of vicine, <1 mg of levodopa, and <0.1 mg of tyramine.

## 1. Introduction

The fava bean (*Vicia faba*) has been identified as a “high-protein crop” suitable for large-scale cultivation as a sustainable plant source of dietary protein [1]. Fava beans contain 24-32% (*w*/*w*) protein, and the fava bean protein (like the protein from soy and other legumes) does not exhibit the human dietary lysine deficiency associated with cereal proteins (Cardador-Martinez et al. 2014, [2]). By virtue of these and additional attributes—including nitrogen fixation capacity and soybean substitution potential—fava beans (with a global production of 4.1 million tons in 2014) are regarded as one of the more globally important legume crops, and research directed at increasing yield, protein, and stress resistance, as well as decreasing antinutritional factors, is underway [2–5].

In addition to the antinutritional factors common to legumes (phytic acid, saponins, polyphenols, and protease inhibitors), fava beans typically contain three additional components of physiological significance: (a) uniquely high levels of vicine (VC) and related pyrimidines (aka alkaloids), (b) the nonproteinogenic amino acid levodopa (LD), aka, L-dopa and L-3,4-dihydroxyphenylalanine, and (c) the tyrosine decarboxylation product and biogenic amine tyramine (TY) [6]. The two pyrimidine glycosides—VC and convicine—and their respective aglycones—divicine and isouramil—when ingested in millimole quantities may cause the acute hemolytic anemia condition known as “favism” [5]. The dopamine precursor LD may induce both powerful therapeutic (“the most efficacious drug” and the “gold standard” for treating Parkinson's disease) and also adverse (gastrointestinal disturbances, hallucinations, dyskinesias, and even favism) effects [7–10]. TY (which, along with histamine, “is well established as the most toxicologically active biogenic amines, due to their relatively low threshold toxic level—‘as low as 6 mg of TY in a 4-hour period'—in addition to the severity of the symptoms they may cause”) has been associated with “peripheral vasoconstriction, increased cardiac output, increased respiration, elevated blood glucose, and release of norepinephrine” [11–15].

Favism can be prevented by decreasing VC consumption, and researchers have identified several means to effect this decrease, including fava bean germination, fermentation, glucosidase treatment, cooking, roasting, autoclaving, protein precipitation, and selective cultivar breeding ([16, 17], Cardador-Martinez et al. 2014, [3, 5, 18–21]). The isolation of fava bean protein by isoelectric precipitation has been shown to be especially effective in lowering the VC presence, with reductions > 99% reported [17, 21]. Similar reductions of LD and TY may be expected to accompany the fava bean protein isolation process, since both LD and TY are (like VC) relatively small, water-soluble molecules, and therefore subject to separation from the major fava bean proteins (MW ≥ 20,000 Da) [21].

In view of these considerations, the objective of the present study was to determine the concentrations of these biologically significant fava bean components—VC, LD, and TY—in a commercial fava bean protein isolate (FBPI) and in a nutritional product (NP) formulated with the FBPI. The determinations were performed by reversed phase HPLC methods that are described below, along with the validation experimentation completed for each method. The experimental FBPI and NP VC, LD, and TY concentrations were then compared to published fava bean concentrations, as a means of verifying the safety of the FBPI-based NP.

## 2. Materials and Methods

### 2.1. Materials

Tyramine hydrochloride and vicine reference materials were obtained from Sigma-Aldrich (St. Louis, MO, USA). The levodopa reference standard was obtained from the United States Pharmacopeia (Rockville, MD, USA). Potassium phosphate monobasic was also obtained from Sigma-Aldrich (St. Louis, MO, USA). 6 M hydrochloric acid (HCl), double distilled, catalog #504, was obtained from GFS Chemicals (Columbus, OH, USA). HPLC grade acetonitrile (ACN) and methanol were obtained from Honeywell Burdick & Jackson (Muskegon, MI, USA). FBPIs were obtained from commercial protein suppliers. The NPs (obtained from Abbott Nutrition, a division of Abbott Laboratories (Chicago, IL, USA)) comprised fava bean protein-based, shelf-stable, oil-in-water emulsions, containing approximately 4% (*w*/*w*) carbohydrate, 2% (*w*/*w*) fat, and 6% (*w*/*w*) protein, and containing both water- and oil-soluble vitamins (A, B, C, D, E, and K), minerals, other micronutrients, buffers, and flavoring agents (vanilla and cocoa powder). The NP carbohydrate included sucrose and fiber, the fat included high oleic safflower oil, and the protein included fava bean and pea.

### 2.2. HPLC Instrumentation and Columns

Direct HPLC determinations of VC, LD, and TY were performed on an Agilent Model 1260 HPLC system (Agilent Technologies, Wilmington, DE, USA), using the equipment and parameters specified in Table 1.

### 2.3. Analyte Extraction Procedures

Since all three analytes (VC, LD, and TY) are relatively small molecules (MW ≤ 304 Da) with appreciable water solubility (≥5 g/L at 20°C; Yalkowsky and Dannenfelser, 1992), the procedures for VC, LD, and TY extraction from FBPIs and NPs were adapted from published procedures developed previously for the extraction of similar relatively small, water-soluble analytes, including methionine, arginine, glutamine, *β*-alanine, *β*-hydroxy-*β*-methylbutyrate, and 5-methyltetrahydrofolic acid [22–25].

### 2.4. Preparation of VC Reference Standard Solutions

A VC reference standard stock solution was prepared by dissolving 1.10–1.50 mg of VC (accurately weighed) in 10 mL of Milli-Q Plus water. A reference standard intermediate solution was prepared by diluting 8.00 mL of the stock solution to 50 mL with Milli-Q Plus water. Reference standard solutions A-E were prepared by pipetting 1.00, 2.00, 3.00, 4.00, and 5.00 mL of reference standard intermediate solution into individual 25 mL volumetric flasks and diluting each to volume with HPLC Mobile Phase A. The five vials obtained by this procedure (reference standard solutions A-E) are used to calibrate each HPLC determination of VC, convicine, divicine, and isouramil. The VC concentrations in the standard solutions range from 0.70-0.96 mg/L (A) to 3.5-4.8 mg/L (E), or 2.3-3.2 *μ*M (A) to 12-16 *μ*M.

### 2.5. Preparation of FBPIs and NPs for the Determination of VC

An aqueous suspension of the FBPI was prepared by (a) transferring 0.80-0.85 g of FBPI (accurately weighed) into a 100 mL glass bottle, (b) adding 100 mL of Milli-Q Plus water and a stir bar to the bottle, (c) capping the bottle, and (d) stirring the mixture vigorously for thirty minutes at room temperature (21°C). An aliquot of the FBPI suspension was centrifuged at 15,000 × *g* for five minutes, and the supernatant was syringe filtered through a 0.45 *μ*m PTFE membrane into an HPLC autosampler vial. The vial was sealed and tested for VC, convicine, divicine, and isouramil by the HPLC system outlined in Table 1, after calibration with reference standard solutions A-E. It should be noted that in the absence of a reference standard for convicine, divicine, and isouramil, their concentrations were estimated from the VC calibration curve. In each case, the UV molar response (peak area at 276 nm) was assumed to approximate the VC UV molar response, so that mass-based concentrations (mg/kg) were calculated using the corresponding MW ratio factor: the convicine mass conversion factor = 305.2/304.3 = 1.003, the divicine mass conversion factor = 142.1/304.3 = 0.467, and the isouramil mass conversion factor = 143.1/304.3 = 0.470.

The NP was prepared by (a) pipetting 10-11 g (accurately weighed) of NP into a 100 mL volumetric flask, (b) diluting to volume with HPLC Mobile Phase A, (c) carefully adding a stir bar and a stopper, and (d) stirring vigorously for thirty minutes at room temperature (21°C). An aliquot of the suspension was centrifuged at 15,000 × *g* for five minutes, and the supernatant was syringe filtered through a 0.45 *μ*m PTFE membrane into an HPLC autosampler vial. The prepared sample was tested for VC by the HPLC system outlined in Table 1, after calibration with reference standard solutions A-E.

### 2.6. Preparation of LD Reference Standard Solutions

A LD reference standard stock solution was prepared by dissolving 10-11 mg of USP LD reference standard (accurately weighed) in 25 mL of Milli-Q Plus water. Low, middle, and high standard solutions were prepared by pipetting 0.0200, 0.100, and 0.200 mL of reference standard stock solution into individual 10 mL volumetric flasks and diluting each to volume with HPLC Mobile Phase A. The three vials obtained by this procedure were used to calibrate each HPLC determination of LD. The LD concentrations in the standard solutions ranged from 0.80-0.88 mg/L (low standard solution) to 8.0-8.8 mg/L (high standard solution), or from 4.1-4.5 *μ*M to 41-45 *μ*M, respectively.

### 2.7. Preparation of FBPIs and NPs for the Determination of LD

The FBPI was prepared for LD determination by (a) weighing 0.9-1.1 g of FBPI into a tared 20 mL glass vial, (b) adding 20.0 mL of Mobile Phase A and a stir bar to the vial, (c) capping the bottle, and (d) stirring the mixture vigorously for thirty minutes at room temperature (21°C). The FBPI suspension was filtered through Whatman No. 41 paper, and the filtrate was syringe filtered through a 0.45 *μ*m PTFE membrane into an HPLC autosampler vial. The vial was sealed and tested for LD by the HPLC system outlined in Table 1, after calibration with the low, middle, and high standard solutions.

The NP was prepared by (a) pipetting 20.0 mL (accurately weighed) of NP into a 50 mL Erlenmeyer flask, (b) adding 20.0 mL of HPLC Mobile Phase A, (c) carefully adding a stir bar and a stopper, and (d) stirring vigorously for sixty minutes at room temperature (21°C). An aliquot of the suspension was centrifuged at 30,000 × *g* and at 20°C for 1 hour, the supernatant was ultrafiltered with a Pall Microsep™ Advance 30K Centrifugal Device, and the ultrafiltrate was syringe filtered through a 0.2 *μ*m PTFE membrane into an HPLC autosampler vial. The prepared sample was tested for LD by the HPLC system outlined in Table 1, after calibration with the low, middle, and high standard solutions.

### 2.8. Preparation of TY Standard Solutions

A TY stock standard solution was prepared by dissolving 28-30 mg of TY HCl reference material (accurately weighed) in 250 mL of Milli-Q Plus water. Low, middle, and high standard solutions were prepared by pipetting 2.00 mL of TY stock standard solution into 2000 mL, 1000 mL, and 500 mL volumetric flasks, respectively, and diluting each to volume with Milli-Q Plus water. The low, middle, and high standard solutions obtained by this procedure were used to calibrate each HPLC determination of TY. The TY concentrations in the standard solutions ranged from 0.089-0.095 mg/L (low standard solution) to 0.35-0.38 mg/L (high standard solution), or from 0.65-0.69 *μ*M to 2.6-2.8 *μ*M, respectively.

### 2.9. Preparation of FBPIs and NPs for the Determination of TY

The FBPI was prepared for TY determination by (a) weighing 0.9-1.1 g of FBPI into a tared 100 mL volumetric flask, (b) diluting to volume with Mobile Phase A (with swirling to thoroughly disperse the FBPI), (c) adding a stir bar and a stopper to the flask, and (d) stirring the mixture vigorously for fifteen minutes at room temperature (21°C). The FBPI suspension was syringe filtered through a 0.45 *μ*m PTFE membrane into an HPLC autosampler vial. The vial was sealed and tested for TY by the HPLC system outlined in Table 1, after calibration with the low, middle, and high standard solutions.

The NP was prepared by (a) pipetting 10.0 mL (accurately weighed) of NP into a 100 mL volumetric flask, (b) diluting to volume with HPLC Mobile Phase A, (c) carefully adding a stir bar and a stopper, and (d) stirring vigorously for fifteen minutes at room temperature (21°C). An aliquot of the suspension was centrifuged at 30,000 × *g* and at 20°C for 30 min, and the supernatant was syringe filtered through a 0.45 *μ*m PTFE membrane into an HPLC autosampler vial. The prepared sample was tested for TY by the HPLC system outlined in Table 1, after calibration with the low, middle, and high standard solutions.

### 2.10. Method Validation Experimentation

Experiments were performed to assess method linearity, precision, accuracy, and selectivity. The purity of the VC reference standard was evaluated by comparing its UV molar extinction coefficient to a published value. Linear response was assessed as standard curve coefficient of determination, intermediate precision was assessed as within-day and/or day-to-day RSD (relative standard deviation, which was calculated as [SD × 100%]/average, where SD is the standard deviation [associated with the analyte concentration average], and average is the average of the experimentally determined analyte concentrations, e.g., the RSD for vicine concentrations of 282, 316, and 319 mg/kg would be calculated as [21 × 100%]/306 = 6.9%, where SD = 21 mg/kg and where the average vicine concentration is 306 mg/kg), accuracy was assessed by known addition recovery experimentation, and analyte selectivity was demonstrated as baseline chromatographic resolution of the analyte from a compound of similar structure (e.g., VC vs. convicine, and TY vs. free tyrosine) and/or by the absence of chromatographic interference in standard and sample reagent blanks. For each analyte (VC, LD, and TY), the limit of detection (LOD) and the limit of quantitation (LOQ) were estimated by “the most widespread approach used in HPLC methods”: by analyte signal comparison to a manually measured standard blank noise basis, where the LOD corresponds to the analyte concentration exhibiting a signal-to-noise (S/N) ratio = 3, and where the LOQ corresponds to the analyte concentration exhibiting a S/N = 10 [26].

## 3. Results and Discussion

### 3.1. VC Method Validation

The UV molar extinction coefficient (13,250 L/mole cm, 275 nm, pH 6.8) of the Sigma-Aldrich VC reference standard was 100.4% of a published value (13,200 L/mole cm, 275 nm, pH 6.8; [27]) and was accordingly regarded as 100% pure. Standard curve linearity was evaluated as the coefficient of determination (*R*^2^) for five VC standard curves (VC concentration range ~0.8 to ~4 mg/L). *R*^2^ exceeded 0.9990 for each of the five plots, and the *y*-intercept averaged 0.9 ± 0.8% (*n* = 5) of the standard solution C (middle standard) peak area.

Precision, as within-day RSD values for a commercial lot of FBPI, was 0.3% (*n* = 3), 0.5% (*n* = 3), and 0.4% (*n* = 3) for days 1, 2, and 3, respectively. The average VC concentration for the series of determinations was 290 ± 4 mg/kg (*n* = 3 days), so that the day-to-day RSD was 1.4%. When three different lots of FBPI obtained from the same supplier were tested, an average VC concentration of 306 ± 20mg/kg was obtained, so that the lot-to-lot RSD was 6.7% (*n* = 3 lots, Table 2).

Two assessments of method accuracy were performed. In the first experiment, a commercial FBPI was prepared for vicine determination by five different procedures, including (a) the sample preparation described above (control), (b) the control procedure with stirring time extended from 30 min to 60 min, (c) the control procedure with temperature increased from 21°C to 75°C, (d) the control procedure using methanol/water (50/50, *v*/*v*) as the extraction medium, and (e) the control procedure using HPLC Mobile Phase A as the extraction medium. Each preparation was tested for vicine concentration, which ranged from 290 to 293 mg per kg FBPI, with an average of 291 ± 1 mg/kg (*n* = 5). There was no significant difference among the five preparations, indicating that an increase in stirring time (b), an increase in extraction temperature (c), an increase in extraction medium hydrophobicity (d), and a decrease in extraction medium pH (e) failed to result in a vicine concentration increase, thereby verifying that vicine was quantitatively extracted by the control procedure (a). In the second experiment, each of the two NPs formulated with an FBPI was tested, without and with VC spiking at 25 mg/kg, for VC. The VC concentrations determined in the unspiked samples were 12.5 and 12.8 mg/kg, and the recoveries of the spiked VC were 100% and 99.9%, respectively. Both experimental assessments support the capacity of the method to accurately determine the vicine concentration in FBPIs and in NPs.

Baseline separation of the pyrimidine glycosides—VC (elution time = 19.3 min, UV max = 276 nm) and convicine (elution time = 20.3 min, UV max = 273 nm)—and baseline separation of their respective aglycones—divicine (elution time = 7.7 min, UV max = 283 nm) and isouramil (elution time = 10.8 min, UV max = 278 nm)—were demonstrated. In the absence of reference materials, the convicine, divicine, and isouramil peaks were identified by their diode array UV spectra. Absence of a VC response in standard and sample blanks was also verified.

The VC LOD and LOQ for a FBPI were experimentally determined to be 3 mg/kg or 10 *μ*moles/kg and 10 mg/kg or 30 *μ*moles/kg, respectively. The VC LOD and LOQ for the NP were 1 mg/kg or 3 *μ*moles/kg and 3 mg/kg or 10 *μ*moles/kg, respectively. Both LOQs were well below the VC concentrations expected to reside in the FBPIs (~300 mg/kg) and in the NPs (~12 mg/kg) of interest.

### 3.2. LD Method Validation

The linearity of the LD response was verified by the strong positive correlation between LD peak area and concentration (*R*^2^ = 1.0000) over the standard curve concentration range (0.8 to 8.0 mg/L). Method precision was assessed as within-day RSD (1.5%) for the triplicate determination of LD in a commercial lot of FBPI (LD average = 13.3 ± 0.2 mg/kg; *n* = 3). Method selectivity was verified by experimental demonstration of the absence of a detectable chromatographic peak at the LD elution time in (a) a standard blank, (b) a sample blank, and (c) a pea protein isolate. The LD method LOQs were 0.5 mg/kg for protein ingredients and 0.05 mg/kg for NPs.

### 3.3. TY Method Validation

The linearity of the TY response was verified by the strong positive correlation between TY peak area and concentration (*R*^2^ = 0.99993) over the standard curve concentration range (0.09 to 0.36 mg/L). Method precision was assessed as within-day RSD (0.4%) for the triplicate determination of TY in a NP (0.671 ± 0.003 mg/kg; *n* = 3). Accuracy was assessed by known addition recovery experimentation: the TY spike recovery averaged 98.5 ± 0.2%, *n* = 3, for a NP spiked (in triplicate) at 0.67 mg TY per kg. Method selectivity was verified by experimental demonstration of the baseline resolution of TY and free tyrosine, and by the absence of a detectable chromatographic peak at the TY elution time in a standard blank and in a sample blank. The TY method LOQs were 0.5 mg/kg for protein ingredients and 0.05 mg/kg for NPs.

### 3.4. VC in FBPIs and NPs

**Figure 1:**
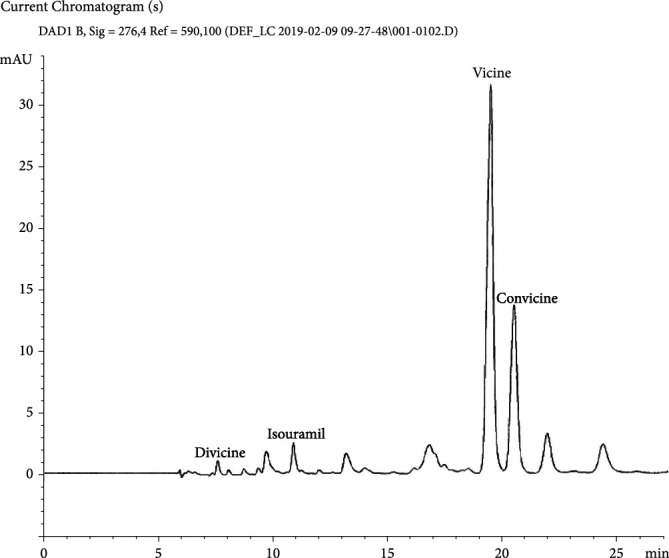
LC/UV chromatogram of a commercial fava bean protein isolate, showing the pyrimidine aglycones divicine and isouramil, and the pyrimidine glycosides vicine and convicine.

The method was applied to three lots of a commercial FBPI (Figure 1). The measured VC concentrations, along with the concentrations estimated for convicine, divicine, and isouramil, are shown in Table 2. The ratio of VC to protein in the FBPI is ~0.034 to 100, *w*/*w*, which is 96-99% lower than the ratio of VC to protein in fava beans (~0.9 to 100 to ~3.2 to 100, *w*/*w*) calculated from published data ([16, 28–30], Cardador-Martinez et al. 2014, [18]). In fact, the reduction approaches the 99 + %VC reduction (fava bean flour to FBPI) achieved by [17]. A VC reduction of this magnitude (96-99%) is not unexpected in view of the capacity of protein concentration processes to separate the relatively small, hydrophilic alkaloids (MW ≤ 305 Da) from the major fava bean proteins (MW ≥ 20,000 Da) [21]. In fact, there are three steps in the FBPI production process that would be expected to remove an appreciable portion of the alkaloids from the protein, namely, the (a) soaking/centrifugation, (b) acid precipitation/centrifugation, and (c) washing/centrifugation steps [31]. In each of the three steps, the aqueous medium would dissolve alkaloids, and these dissolved alkaloids would then be separated from the protein by the subsequent centrifugation. An analogous reduction/removal of soluble sugars and isoflavones (including a preferential reduction of the more soluble isoflavone glucosides vs. the less soluble isoflavone aglycones) has been shown to occur in similar processes used in the production of soy protein isolate from soy protein flour, wherein “washing was the step where most isoflavones were lost” [32]. It is also relevant to note that the soy isoflavones are both larger and less water-soluble than VC, LD, and TY (e.g., the major soy isoflavones daidzin and genistin have MW = 416 and 432 Da, and water solubility = 0.661 and 1.01 g/L, respectively [33, 34]), so that the expectation is that VC, LD, and TY (MW ≤ 304 Da, water solubility ≥ 5 g/L) would be more readily removable than the soy isoflavones. The expectation for extensive VC, LD, and TY reduction during the FBPI production process is further supported by extensive reductions of cresol sulfates and indoxyl sulfate (>90% reduction) and of lactose (>99% reduction) that occur during the production of milk protein concentrates/isolates from milk [35, 36].

The method was also applied to two NPs, which were found to contain VC at 12.5 and 12.8 mg/kg (corresponding to 0.0219 and 0.0224 g/100 g protein, respectively). These VC concentrations were 77.2% and 79.0%, respectively, of the VC concentration that was projected from its FBPI ingredient, indicating a processing-induced VC loss of 21-23%. VC losses in this range are consistent with boiling (average VC loss = 19% ± 12%, *n* = 10), stewing (VC + convicine loss = 21.6%), cooking (VC loss = 35%), and autoclaving (VC loss = 37%) losses reported in published studies [16, 37–39].

Based on the alkaloid sum estimated for the FBPI (1.36 *μ*mole/g, with molar distribution = 74/20/2/4 VC/convicine/divicine/isouramil), a maximum alkaloid sum of 76 *μ*moles per kg of NP may be calculated. This estimated alkaloid concentration is ~300× lower than the corresponding alkaloid estimate (20,000-30,000 *μ*moles per kg) calculated for raw fava beans ([29, 30], Khalil et al., [28], Cardador-Martinez et al. 2014, [18]), a difference that is relevant since most cases (“>96%”) of favism are associated with the consumption of raw beans (suggesting that the incidence of favism is significantly decreased even by the 20-40% alkaloid loss and/or by the fava bean *β*-glucosidase inactivation induced by cooking) and the fact that the consumption of “a large meal of low-vicine fava beans [the VC was 10-20x lower than that in typical fava beans]” resulted in no favism [40, 41]. A formal evaluation of these safety issues has been documented in a FBPI GRAS application [31].

### 3.5. LD in FBPIs and NPs

**Figure 2:**
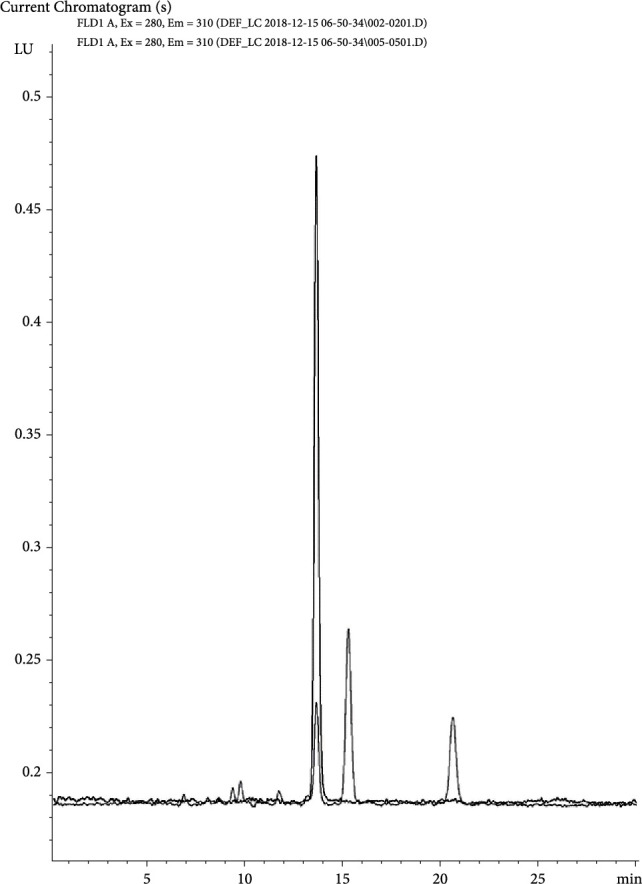
Superimposed LC/UV chromatograms of a levodopa standard solution (with the taller levodopa peak at 13-14 minutes) and a commercial fava bean protein isolate (with the shorter levodopa peak at 13-14 minutes).

The commercial FBPI was found to contain LD at 13.3 ± 0.2 (*n* = 3) mg per kg or 1.5 mg of LD per 100 g of protein (Figure 2). Comparing this to a LD maximum concentration of 0.6 g per kg of dried fava beans [6, 42], and assuming a protein content of 300 g per kg of dried beans, the fava bean LD concentration may be estimated at 200 mg per 100 g protein. It is worth noting that much higher LD concentrations (>10× higher) have been reported for undried fava bean seeds [42–46]. The FBPI protein-based LD concentration would therefore be ~99% reduced vs. the fava bean protein-based LD concentration, a reduction comparable to the corresponding reduction of VC.

The LD concentrations in the NPs were <0.05 mg/kg (vanilla) and 0.17 mg/kg (chocolate), which correspond to <0.1 mg/100 g protein (vanilla) and 0.3 mg/100 g protein (chocolate). The NP protein-based LD concentrations are therefore in the range of 1000× lower than the published fava bean protein-based LD average concentration (~200 mg/100 g protein).

As indicated above, the substantial depletion of LD in the FBPI and in the NPs formulated with it is not unexpected, in view of LD's (a) relatively small size (197.2 Da), (b) aqueous solubility (octanol/water partition coefficient *X*Log*P*3 = −2.7), and (c) instability to neutral/alkaline pH and heat ([7, 9]; PubChem 2020). These attributes render LD vulnerable to large losses during FBPI and NP processing steps (e.g., washing/centrifugation and sterilization).

### 3.6. TY in FBPIs and NPs

The commercial FBPI did not contain a quantifiable level of TY, meaning that the TY concentration was <0.5 mg per kg or<0.06 mg of TY per 100 g of protein. Moret et al. [13] measured TY at 10 mg per kg or ~4 mg per 100 g of protein. When expressed on a protein basis, the FBPI protein-based TY concentration is in the range of 98-99% lower than the published fava bean TY concentration [13].

**Figure 3:**
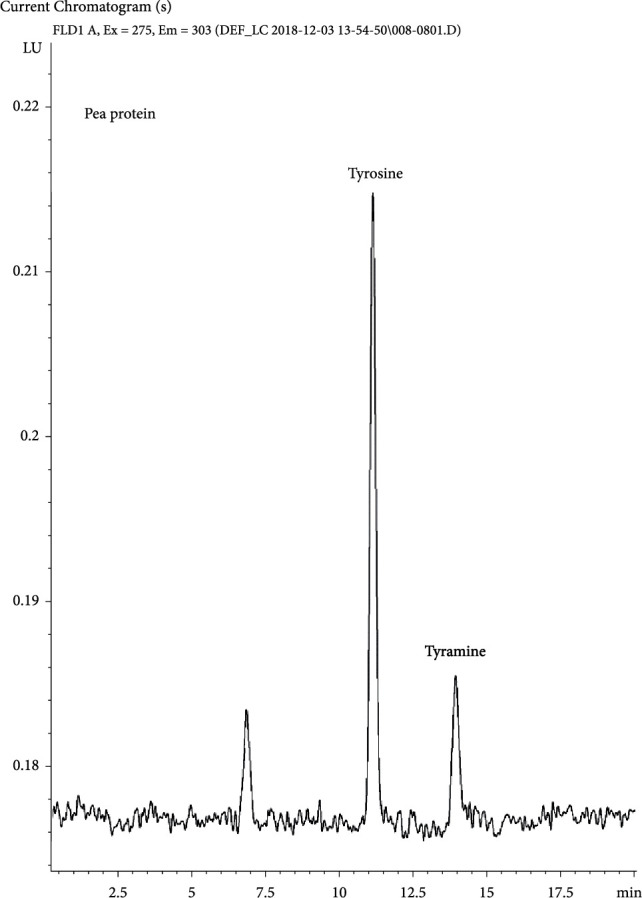
LC/FLD chromatogram of the pea protein ingredient, showing tyrosine and tyramine.

The TY concentration in both NPs was 0.08 mg/kg, which corresponds to 0.14 mg/100 g protein. The NP protein-based TY concentration was therefore <4% of the published fava bean protein-based TY concentration (~4 mg/100 g protein). It is worth noting that TY was found at ~0.5 mg/100 g protein in the NP's pea protein ingredient (Figure 3), which provides 17% of the total protein in the NP formulation, thereby accounting for the majority (>60%) of the TY in the NPs (0.08 mg/kg).

Finally, as indicated above, the substantial TY depletion in the FBPI (vs. the published fava bean TY concentration) is presumably a consequence of the small size (137 Da) and the water solubility (10.4 g/L at 15°C [47]) of the TY molecule, facilitating its elimination during FBPI production.

## 4. Conclusions

A commercial FBPI was found to contain VC at 306 mg/kg, LD at 13.3 mg/kg, and TY at <0.5 mg/kg. When expressed on a protein basis, these concentrations (34, 1.5, and <0.06 mg per 100 g protein, respectively) are at least 96% lower, ~99% lower, and at least 98% lower than the VC, LD, and TY concentrations reported for fava beans (when also expressed on a protein basis: ≥900 mg, ~200 mg, and ~4 mg per 100 g protein, respectively). The NP formulated with the fava bean protein isolate contained VC at 13 mg/kg, LD at ≤0.17 mg/kg, and TY at 0.08 mg/kg. When expressed on a protein basis, these concentrations (22, ≤0.3, and 0.14 mg per 100 g protein, respectively) are 97% lower, >99% lower, and 96% lower than the fava bean (protein-based) VC, LD, and TY concentrations. The corresponding concentrations delivered by one serving (11 fl. oz.) of the NP would therefore be approximately 5 mg of VC, <1 mg of LD, and <0.1 mg of TY.

## Figures and Tables

**Table 1:** HPLC instrumentation and parameters.

Analyte(s)	Vicine, convicine, divicine, and isouramil in FBPI	Vicine in NP	Levodopa	Tyramine
LC system	Agilent Model 1260 (Agilent Technologies, Wilmington, DE, USA)			
Detector	Agilent G4212B diode array		Agilent G1321A fluorescence	
Detection wavelengths	276 nm, 214 nm, 273 nm, 278 nm, 283 nm	276 nm, 214 nm	Ex = 280 nm Em = 310 nm	Ex = 275 nm Em = 303 nm
LC column	YMC-Pack ODS-AQ^a^	Zorbax Eclipse Plus C18^b^	Zorbax Eclipse Plus C18^b^	YMC-Pack ODS-AQ^a^
Temperature	25°C	15°C	20°C	15°C
Mobile phase A	0.05 M KH_2_PO_4_, pH 2.9		1000/25 (*v*/*v*) 0.05 M KH_2_PO_4_, pH 2.9/ACN	0.02 M KH_2_PO_4_, pH 2.9
Mobile phase B	200/800 (*v*/*v*) H_2_O/ACN			
Flow rate	0.5 mL/min	0.4 mL/min	0.4 mL/min	0.5 mL/min
Injection	10 *μ*L	10 *μ*L	5 *μ*L	10 *μ*L
Elution program	0% B 0-20 min, 100% B 20-25 min, 0% B 25-45 min (end)	0% B 0-25 min, 100% B 25-30 min, 0% B 30-45 min (end)	0% B 0-25 min, 100% B 25-30 min, 0% B 30-50 min (end)	0% B 0-5 min, 0-10% B 5-25 min (linear), 100% B 25-30 min, 0% B 30-45 min (end)

^a^4.6 × 250 mm, 5 *μ*m, 120 Å; Waters Corporation, Milford, MA, USA. ^b^4.6 × 250 mm, 5 *μ*m; Agilent Technologies, Wilmington, DE, USA.

**Table 2:** Pyrimidine concentrations, as mg/kg, in a commercial fava bean protein isolate.

Alkaloid	Lot 1	Lot 2	Lot 3	Average (RSD; *n* = 3 lots)
Vicine	282	316	319	306 ± 21 (6.7%)
Convicine^∗^	66	91	86	81 ± 13 (16%)
Divicine^∗^	4	4	4	4 ± 0 (0%)
Isouramil^∗^	5	10	10	8 ± 3 (40%)
Sum	357	421	419	399 ± 36 (9.0%)

^∗^The concentrations of convicine, divicine, and isouramil were estimated using the vicine standard curve (with adjustment for MW differences).

## Data Availability

All supporting data are included in the manuscript.

## Supplementary Materials

Supplementary Materials Graphical abstract. The vicine, levodopa, and tyramine concentrations (relative to the fava bean concentrations) in a commercial fava bean protein isolate and in a nutritional product formulated with the commercial isolate. All concentrations are expressed as % of the fava bean concentration, and all concentrations are expressed on a protein basis (mass per unit protein).
