# Supplementary material for: Substantial Depletion of Vicine, Levodopa, and Tyramine in a Fava Bean Protein-Based Nutritional Product
Source: Int J Food Sci. 2021 Jan 29;2021:6669544. doi: 10.1155/2021/6669544 (PMC7867441; doi:10.1155/2021/6669544)
Supplement: Supplementary file 1 [file 6669544.f1.docx]

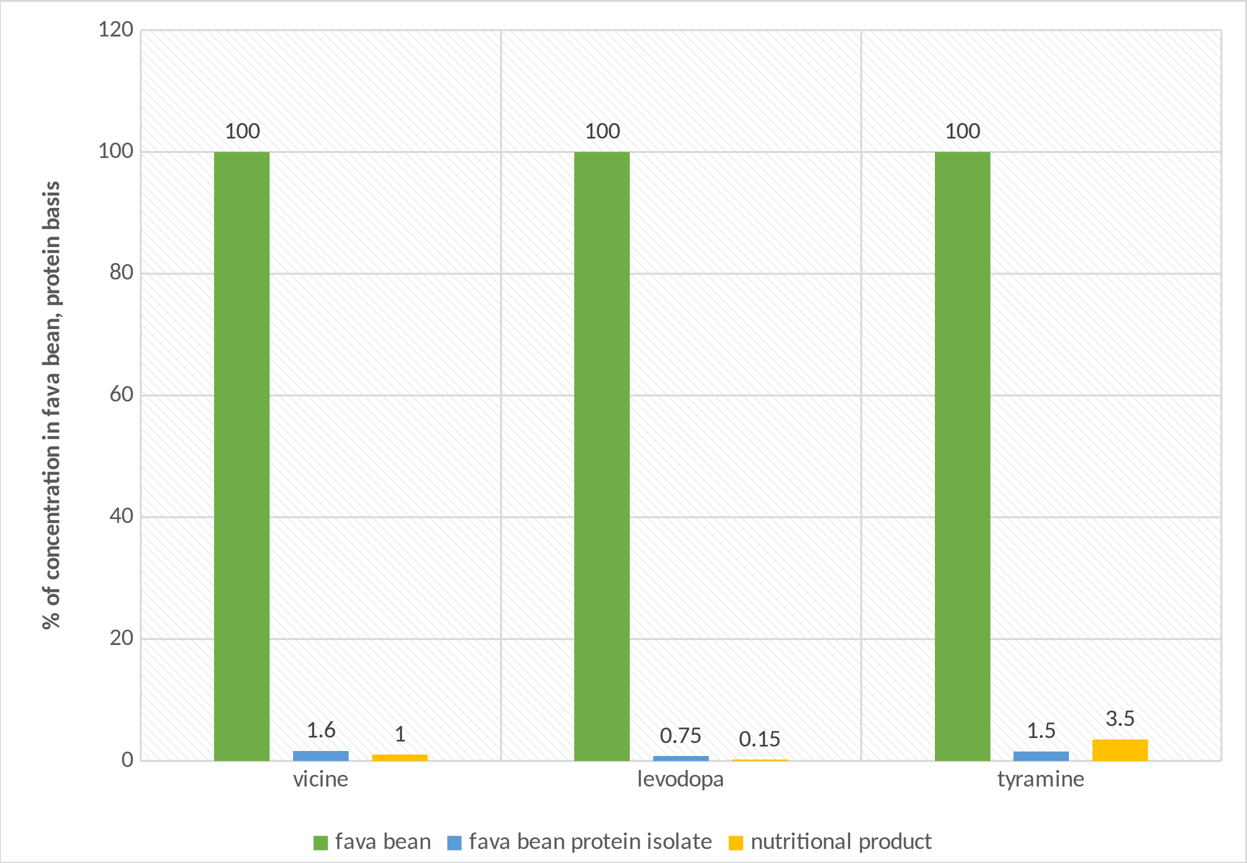
120
100
80
60
40
20
0
% of concentration in fava bean, protein basis
100
1.6
1
100
0.75
0.15
100
1.5
3.5
vicine
levodopa
tyramine
fava bean
fava bean protein isolate
nutritional product
